# Assessing the Productivity of Colonies Headed by Preheated Honeybee Queens

**DOI:** 10.3390/insects16080858

**Published:** 2025-08-18

**Authors:** Abd Al-Majeed Al-Ghzawi, Shahera Talat Zaitoun, Mohammad Nafi Solaiman Al-Sabi, Salem Saleh Mazari, Ilham Mustafa Al-Omari, Maqbool Saed Altalhi

**Affiliations:** 1 Department of Plant Production, Faculty of Agriculture, Jordan University of Science and Technology, P.O. Box 3030, Irbid 22110, Jordan; emamassad@gmail.com; 2 Department of Plant Production & Protection, Faculty of Agricultural Technology, Al-Balqa Applied University, P.O. Box 206, Salt 19117, Jordan; zaitoun@bau.edu.jo; 3 Department of Basic Medical Veterinary Sciences, Jordan University of Science and Technology, P.O. Box 3030, Irbid 22110, Jordan; mnalsabi@just.edu.jo; 4 Ministry of Agriculture, P.O. Box 2099, Amman 11181, Jordan; ssmazarijo@outlook.com; 5 Taif Beekeepers Cooperative Society, TIDA2375, Taif 26724, Saudi Arabia; maqbool81@gmail.com

**Keywords:** beehives productivity, flight performance, honeybee behavior, preheat hardening, climate change effects, environmental stressors

## Abstract

This study investigated the effects of preheat hardening on the egg-laying capacity of honeybee queens and the flight performance of their daughter workers. A honeybee queen was confined in a cage with a two-section frame for 12 h. Then, 48 h old eggs from one section were incubated for 15 min at 41 °C and 70% relative humidity (RH). The queens (n = 12) raised in this section were named the pre-heat-treated queens (pH-TQs). Eggs from the second section were exposed to 34.5 °C and 70% RH for 15 min, and the queens raised in this section were named the non-heat-treated queens (nH-TQs) (n = 12). After mating, both groups were introduced to queenless hives in order to produce workers. The results show that, during the study period (2021 and 2022), the colonies headed by the pH-TQs reared significantly more brood cells and worker adults (especially during the summer), collected and stored more pollen, had less tendency to rear drone brood, and constructed fewer swarm cells than the colonies headed by the nH-TQs. Preheat hardening can be a promising method for improving honeybee workers’ reproductive potential and adaptability, allowing for better adaptation to environmental changes, thus compensating for the mass fatalities of honeybees globally.

## Simple Summary

Preheat hardening in the immature stages causes marked changes in the morphology of honeybee workers. In this study, we investigated the effects of such preheat hardening on the reproductive capacity of honeybee queens and the subsequent productivity of the workers. We found that heat-treated queens produced workers that had significantly increased numbers of brood cells and worker adults, especially during the hot summer months. These workers also managed to collect and store more pollen, had less tendency to rear drone brood, and constructed fewer swarm cells than non-heat-treated workers. We therefore propose the use of heat hardening to compensate for the current loss of honeybees globally.

## 1. Introduction

In recent decades, heat has been increasingly used for the control of various pests causing economic harm, as well as for the control of food and non-food items in stores [1,2,3], plant diseases [4], human therapy [5], Varroa mites inside hives [6,7], certain honeybee fungal diseases [8], and viral diseases [9]. In a more advanced step of utilizing heat, several studies have demonstrated that the thermal manipulation of honeybee queens with non-lethal heat shock during the egg stage has beneficial effects on the subsequent physiology and behavior of their daughter workers. This is defined as rapid heat hardening and has been practiced to improve the survival of bees [10,11]. Honeybees show a marked response to heat exposure in terms of physiology [12], including changes in antioxidant and detoxification enzymes [13]; adjustments in metabolism and nutrient supply [14,15]; an increase in gene expression levels when exposed to heat, cold, and starvation stress [16,17,18]; and, in queens and workers, even a change in color [19]. In terms of the behavioral responses to heat exposure [20,21], honeybees also show changes in their development [22], immunity [23], phenology [24], ability to learn [20,25], behavioral performance [26,27], disease susceptibility [28], and flight behavior [21,29].

To overcome temperature fluctuations and prevent extremely high temperature levels inside the hive, honeybee workers in the colony strictly keep it within a range of 33–36 °C. This is because the brood is very sensitive to changes in the hive temperature [30]. It has been previously reported that the danger of overheating is mitigated by behavioral changes in the workers, including the activation of fanning behavior, i.e., rapid vibration of the wings [31]; collecting and depositing water inside the hive to allow for evaporative cooling [32]; relocating bees outside the entrance to facilitate continuous ventilation and evaporation inside the hive [33]; and shielding honey and brood combs from sources of external heat [34]. These and other behaviors have allowed the honeybee subspecies to successfully live in a wide range of climates, from hot and dry to tropical and temperate climates.

To the best of our knowledge, the effects of the heat shock treatment of honeybee queens during the developmental stages on brood rearing and worker populations have not yet been investigated. We hypothesized that the exposure of honeybee queens to non-lethal heat shock during the egg stage could improve their viability and workers’ honey production. The results of this study help determine whether exposure to high temperatures during the developmental stages adjusts the queen’s egg-laying capacity. Furthermore, they provide a scientific basis for further exploration of the temperature adaptation mechanism of queen honeybees, ultimately helping to formulate a comprehensive improvement program.

## 2. Materials and Methods

### 2.1. Queen Rearing and Thermal Manipulation

The origin of the experimental colonies (n = 24) was healthy honeybees headed by the local race, the Syrian honeybee (*Apis mellifera* syriaca), selected from a privately owned apiary in northern Jordan. The colony queens were reared in April 2020, which is known to be the prime month for open mating in the Jordan Valley [35]. A queen from a colony was confined in a queen excluder with a two-section frame for 12 h to produce eggs of approximately the same age. Just before egg hatching (48 h old), both sections were taken from the colony after brushing the nurses off, and they were transferred to the lab at Agri-JUST. The first section was incubated at 41 °C and 70% relative humidity (RH) for 15 min and then returned to its mother hive. After egg hatching, the first-instar worker larvae were grafted to queen cups to be raised in a queenless hive, following standard apicultural procedures [36]. The queens raised under this treatment were named the pre-heat-treated queens (pH-TQs). The second section was exposed to 34.5 °C and 70% RH for 15 min and then returned to its mother hive. After egg hatching, the same procedure was followed to rear non-heat-treated queens (nH-TQs) as the control. Just before pupal emergence, plastic mesh cages were fitted over the occupied pupal queen cells of both groups to retain the emerging virgin queens. The emerging virgin queens in both groups were removed from the plastic mesh cages and introduced to queenless mating mini-hives. These hives were inspected two weeks later to ensure the success of the introduction and the onset of oviposition. From each treatment group, 12 queens were introduced to queenless experimental colonies following standard procedures [36]. Six months later, the experimental colonies were well established, occupying 8–10 frames. Before the commencement of the study in November, each colony was treated with amitraz against Varroa mites, and all experimental colonies were equalized to include a similar amount/number of stored food, brood, and adult bees.

During the investigation, the colonies were subjected to various apiculture practices, including shading, feeding, supering, and external pest control, as needed. The same procedure was repeated to produce new colonies headed by new queens treated in the same manner as above for use in the second year of the study.

### 2.2. Apicultural Practices

As usually practiced by beekeepers in Jordan, from November to April, the experimental colonies were placed on a designated farm in Shuna North in the Jordan Valley (32°37′′ N, 35°36′′ E), which is 200 m below sea level. Throughout this period, warm weather conditions prevailed in the Jordan Valley, together with the early flowering of cultivated and wild plants. As the temperatures increased and the wild and cultivated plants dried out in the Jordan Valley, the experimental colonies were relocated to the highlands in an apiary located in the Karak district (31°16′′ N, 35°28′′ E), with an elevation of 920 m above sea level. The same practices were repeated in the second year of the study. To reduce bee drifting, the locations of the hives were selected to ensure good protection from the wind. A total of 24 hives headed by pH-TQs and nH-TQs (n = 12 each) were randomly placed in each location in two rows; the rows were spaced four meters apart, while the hives in each row were spaced two meters apart.

### 2.3. Measurement of Worker and Drone Brood Areas

To estimate the cumulative worker and drone production and emergence patterns in a colony, the sealed brood area of each caste was measured throughout the season. This was carried out by placing a clear plastic grid marked out in 5 × 5 cm squares over the brood on one side of each frame and estimating the number of sealed cells in each quadrat [37].

### 2.4. Count of Adult Workers

The numbers of worker bees in the pH-TQ and nH-TQ colonies were counted at 21-day intervals throughout the experiment using the procedures described by Gerig [37]. The same counting method was employed in visual estimations of the numbers of brood, drone brood, and stored pollen cells and adult workers per unit area. When two counts were recorded in the same month, the average was calculated.

### 2.5. Count of Swarm Queen Cells

All pH-TQ and nH-TQ colonies were inspected at 10-day intervals throughout the swarming period. Queen cups and swarm queen cells with laid eggs or larvae in brood chambers were counted and destroyed. This was carried out between February and May during the study period from 2021 to 2022.

### 2.6. Reporting Pollen Foraging Percentage per Hour

The number of foragers returning to the entrance of each experimental colony was counted for a one-minute duration on an hourly basis between 06.00 AM and 4.00 PM for one day every two weeks. Pollen foragers were defined as workers returning to their colonies with a pollen load, while non-pollen foragers were defined as workers returning with no pollen load [38]. The pollen foraging percentage of a colony was calculated by dividing the number of returning bees with a pollen load by the total number of returning worker foragers to their hives and multiplying by 100.

### 2.7. Count of Stored Pollen Cells

The amount of pollen stored inside the colonies was measured using the same procedure used for bee and brood counting [37].

### 2.8. Weight of Pollen Loads

Pollen samples were collected using pollen traps fitted to the entrances of both the pH-TQ and nH-TQ colonies for one day every two weeks. The accumulated pollen was removed, cleaned, and dried at room temperature. Ten pollen loads from each hive were randomly selected from each pollen trap on each sampling date. The weight of each pollen load was obtained to the nearest 0.1 milligram (mg) using a technical balance (Scientech SP 350, Scientech, Boulder, CO, USA).

### 2.9. Statistical Analysis

All statistical analyses were performed using the R statistical system, Version 4.4.3 [39]. Data are expressed as means ± standard deviation (SD). A repeated-measures ANOVA with a generalized eta-squared test and the F statistic was employed to compare different parameters in all treatment groups. Each treatment was replicated twelve times. Differences were considered significant at *p* < 0.05.

## 3. Results

### 3.1. Brood-Rearing Activities

**Figure 1:**
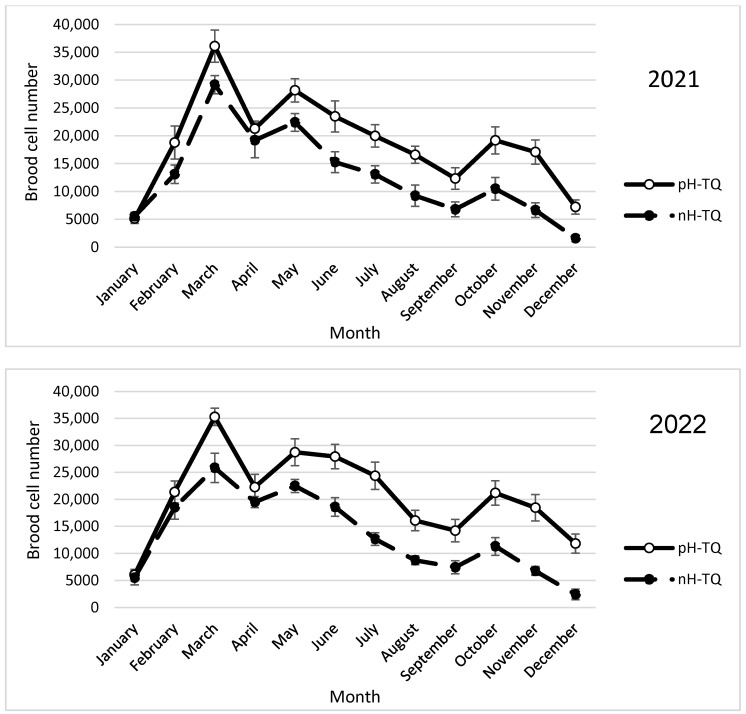
Number of worker brood cells (±standard deviation) of heat-treated honeybee queens (nH-TQ) and thermally manipulated honeybee queens in their immature stages (pH-TQ) in two consecutive years, 2021 and 2022.

The colonies headed by the pH-TQs and nH-TQs showed a triple cycle of brood cell numbers within the two study years of 2021 and 2022 (Figure 1). Both colony groups started brood rearing based on the availability of nectar and pollen plants and the suitability of weather conditions in the Jordan Valley, reaching the entire season’s maximum peak in March (35,700 and 27,512 cells, respectively). The pH-TQ colonies reared significantly more brood areas (*p* < 0.001) than the nH-TQ colonies during the steep rise in brood rearing between February and March. After the colonies migrated to the highlands, where the air temperature and wildflowers were suitable, the pH-TQ colonies showed a significantly higher (*p* < 0.001) second brood peak than the nH-TQ colonies in May (28,454 and 22,454 cells, respectively). The critical period for beekeeping started with the increase in air temperature and the drying of wildflowers by June, and, thereafter, both colony groups showed a decrease in brood-rearing activities. The pH-TQ colonies were less affected by high-temperature stress, and they reared significantly larger (*p* < 0.001) brood areas than the nH-TQ colonies from May to the end of the season. In the autumn, when the air temperature was suitable and certain fruit trees began to flower, the pH-TQ colonies showed a third brood peak with a significantly larger (*p* < 0.001) brood area than the nH-TQ colonies (20,191 and 10,891 cells, respectively). A repeated-measures ANOVA showed a significant difference in the number of brood cells between the pH-MQ and nH-MQ groups (F (1, 574) = 147.10, *p* < 0.001).

### 3.2. Honeybee Worker Population

**Figure 2:**
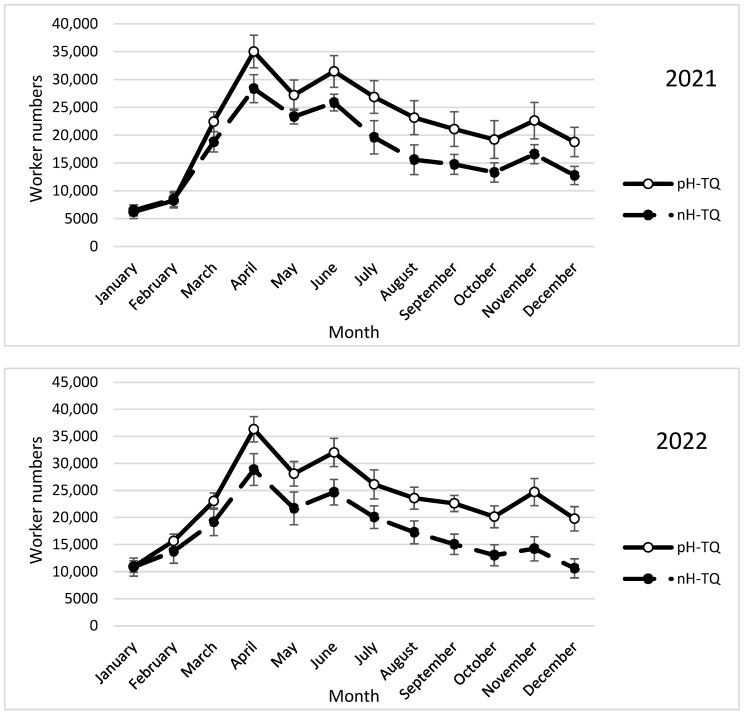
Number of honeybee workers (±standard deviation) of non-heat-treated honeybee queens (nH-TQ) and thermally manipulated honeybee queens in their immature stages (pH-TQ) in two consecutive years: 2021 and 2022.

The annual cycle of the bees corresponded to the rate of brood rearing, and the maximum number of adults lagged behind the maximum rate of brood rearing. Both groups (the pH-TQ and nH-TQ colonies) showed a triple cycle of the worker population during the two study years of 2021 and 2022 (Figure 2). The pH-TQ colonies produced a significantly larger (*p* < 0.001) worker population than the nH-TQ colonies in April (35,033 and 28,366 workers, respectively) and in June (31,466 and 25,866 workers, respectively). During the summer, the pH-TQ colonies showed a steep reduction in worker numbers, while the nH-TQ colonies showed a sharp reduction in their worker numbers. The pH-TQ colonies showed a higher heat tolerance during the summer months and maintained significantly higher (*p* < 0.001) worker numbers throughout the period from June to the end of the year. After the cessation of the hot summer months, both groups of honeybee colonies, headed by the pH-TQs and nH-TQs, showed a triple peak in the worker population, with a significantly larger (*p* < 0.001) population in the pH-TQ colonies than in the nH-TQ colonies (22,625 and 16,600 cells, respectively). The seasonal fluctuations in brood-rearing activities and worker bee populations were fairly constant in terms of both time and relative magnitude throughout the two years of the study. The brood-rearing performance and population dynamics of the adult bees tended to follow the same general cycles in the second year of the study (Figure 2). A repeated-measures ANOVA showed a significant difference in the monthly changes in worker numbers between the pH-MQ and nH-MQ groups (F (1, 574) = 47.68, *p* < 0.001).

### 3.3. Drone Brood Rearing

**Figure 3:**
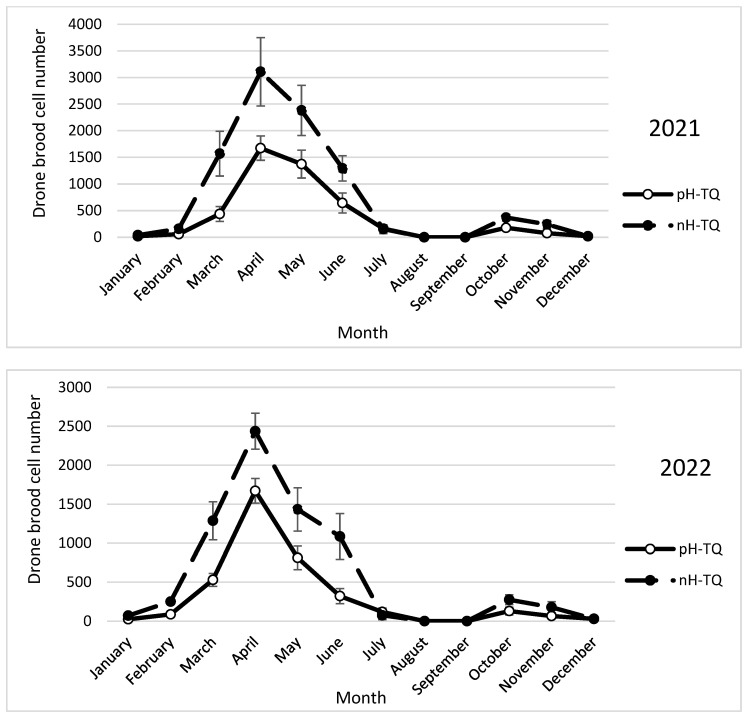
Drone brood cell numbers (±standard deviation) of non-heat-treated honeybee queens (nH-TQ) and thermally manipulated honeybee queens in their immature stages (pH-TQ) in two consecutive years: 2021 and 2022.

The colonies headed by the pH-TQ and nH-TQ queens showed a single cycle of drone brood cell numbers within the two study years of 2021 and 2022 (Figure 3). In both groups of colonies, drone brood rearing began in February and ceased in June. The nH-TQ colonies reared significantly more (*p* < 0.001) drone brood cells (310.3 cells) than the pH-TQ colonies (1675.0 cells in 2021). A repeated-measures ANOVA showed a significant difference in the number of drone brood cells between the pH-MQ and nH-MQ groups (F (1, 574) = 102.13, *p* < 0.001).

### 3.4. Swarm Cells

**Figure 4:**
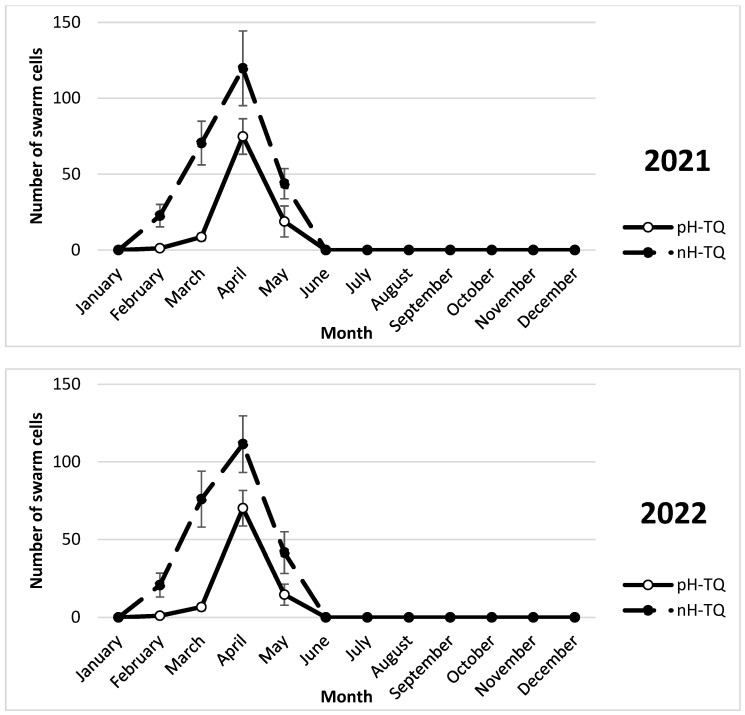
Number of swarm cell constructions (±standard deviation) of non-heat-treated honeybee queens (nH-TQ) and thermally manipulated honeybee queens in their immature stages (pH-TQ) (n = 12 colonies for both groups) in two consecutive years: 2021 and 2022.

The pH-TQ and nH-TQ colonies showed swarm cell construction between March and May of each year of the study period (Figure 4). In the first year of the investigation, the nH-TQ colonies constructed significantly (*p* < 0.001) more swarm cells in April (120 cells) than the pH-TQ colonies (74.8 cells). A repeated-measures ANOVA showed a significant difference in the number of swarm queen cells between the pH-MQ and nH-MQ groups (F (1, 574) = 139.81, *p* < 0.001).

### 3.5. Pollen Foraging Percentage per Hour

**Figure 5:**
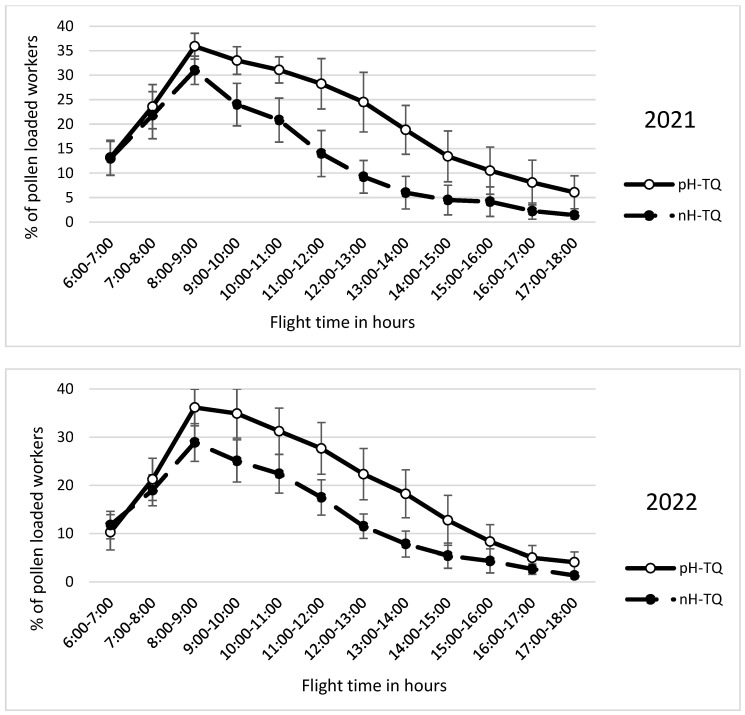
Percentage (%) of pollen loaded workers per hour (±standard deviation) of non-heat-treated honeybee queens (nH-TQ) and thermally manipulated honeybee queens in their immature stages (pH-TQ) in two consecutive years: 2021 and 2022.

The workers in both colony groups, headed by the pH-TQs and nH-TQs, started foraging very early in the day at six o’clock (Figure 5). The pH-TQ colonies showed a significantly (*p* < 0.001) higher foraging rate from 8 AM to the end of the day than the nH-TQ colonies. A repeated-measures ANOVA showed a significant difference in the hours of flight time between the pH-MQ and nH-MQ groups (F (1, 574) = 23.96, *p* < 0.001).

### 3.6. Stored Pollen Cells

**Figure 6:**
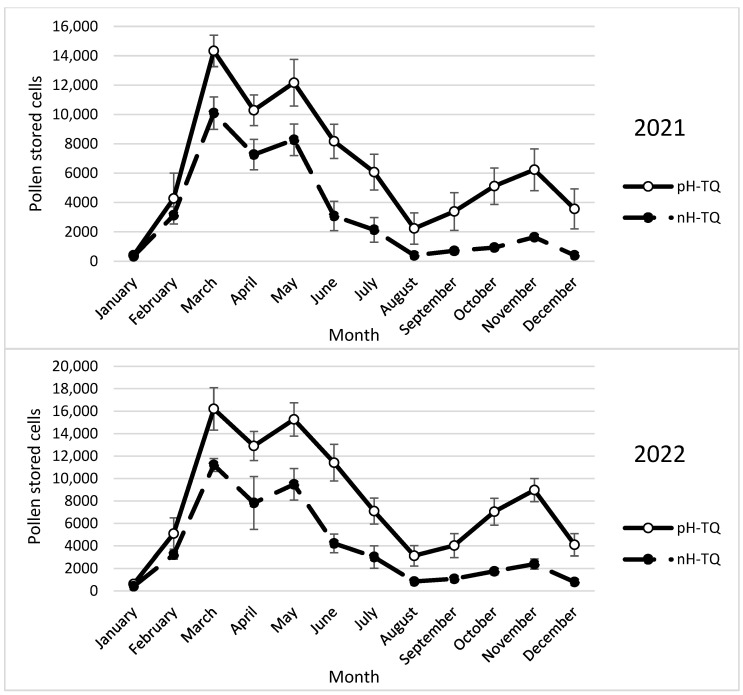
Number of stored pollen cells (±standard deviation) by non-heat-treated honeybee queens (nH-TQ) and thermally manipulated honeybee queens in their immature stages (pH-TQ) (n = 12 colonies for both groups) in two consecutive years: 2021 and 2022.

The number of stored pollen cells in both the pH-TQ and nH-TQ colonies fluctuated between the months of the year (Figure 6). The pH-TQ colonies stored significantly (*p* < 0.001) more pollen cells than the nH-TQ colonies in March (14,350 and 10,100 workers, respectively) and in May (12,167 and 8.275 cells, respectively). The pH-TQ colonies were more tolerant of high-temperature stress than the nH-TQ colonies, showing more active flying outside the hives and storing significantly more pollen cells (*p* < 0.001) during the summertime between May and August. In the autumn, when the air temperatures were more suitable and certain fruit trees began to flower, the pH-TQ colonies again stored significantly more pollen cells (*p* < 0.001) than the nH-TQ colonies (6238 and 1638 cells, respectively). In both colony groups, pollen storage was the lowest between December and February, when cold weather prevailed. A repeated-measures ANOVA showed a significant difference in the number of stored pollen cells between the pH-MQ and nH-MQ groups (F (1, 574) = 64.93, *p* < 0.001).

### 3.7. Monthly Percentage of Pollen Foragers

**Figure 7:**
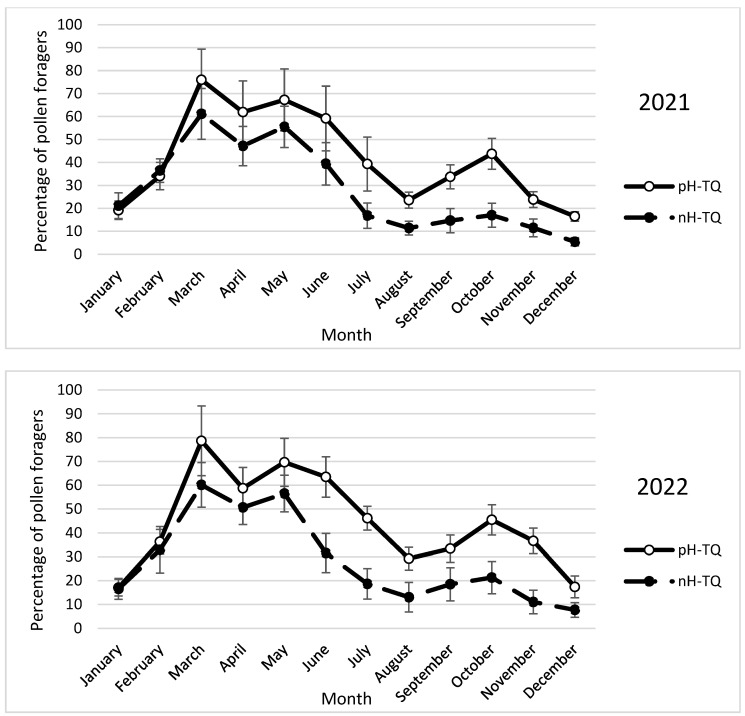
Percentage of returning workers collecting pollen (±standard deviation) in non-heat-treated honeybee queens (nH-TQ) and thermally manipulated honeybee queens in their immature stages (pH-TQ) in two consecutive years: 2021 and 2022.

Workers in both the pH-TQ and nH-TQ colonies tended to collect pollen throughout the two years of the study (Figure 7). In both colony groups, the highest percentage of returning workers, reflecting the flying activity, was observed in March and June of each year. During the period from May to the end of the year, the average percentage of returning workers loaded with pollen grains in the pH-TQ colonies was significantly (*p* < 0.001) higher than that in the nH-TQ colonies. The forager percentages in the pH-TQ colonies were significantly (*p* < 0.001) higher in March, May, and October (76, 67.2, and 31.5%, respectively) than those in the nH-TQ colonies (61.2, 60.1, and 17.0%, respectively). A repeated-measures ANOVA showed a significant difference in the monthly pollen forager percentages between the pH-MQ and nH-MQ groups (F (1, 574) = 31.88, *p* < 0.001).

### 3.8. Weight of 10 Pollen Loads

**Figure 8:**
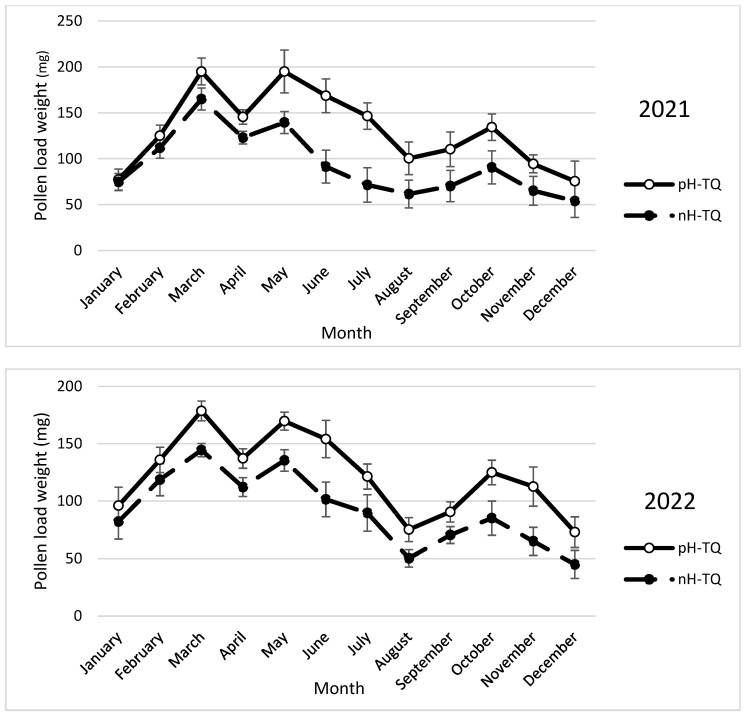
Average weight of 10 pollen loads in milligrams (mg) (±standard deviation) carried by workers of non-heat-treated honeybee queens (nH-TQ) and thermally manipulated honeybee queens in their immature stages (pH-TQ) in two consecutive years: 2021 and 2022.

The average weight of 10 pollen loads carried by the workers in the pH-TQ colonies was significantly heavier than that carried by the workers in the nH-TQ colonies between the period from May to the end of the year (Figure 8). The heaviest pollen loads were recorded from March to May for both the pH-TQ and nH-TQ colonies. The workers in the pH-TQ colonies carried significantly (*p* < 0.001) heavier pollen loads in March, May, and October 2021 (195.1, 195, and 114.2 mg, respectively) than those in the nH-TQ colonies (165.1, 139.5, and 68.3 mg, respectively). A repeated-measures ANOVA showed a significant difference in the pollen load weight between the pH-MQ and nH-MQ groups (F (1, 574) = 48.05, *p* < 0.001).

In the second year of the study (2022), the trends in brood rearing, worker population, pollen collection and storage, swarm cell construction, and drone brood rearing in the pH-TQ and nH-TQ colonies tended to be the same and were consistent in terms of time (Figure 1, Figure 2, Figure 3, Figure 4, Figure 5, Figure 6, Figure 7 and Figure 8). Supplementary Materials can be found in the link provided at the end of this article.

## 4. Discussion

### 4.1. Effect of Preheat Hardening on the Survival of Honeybee Workers

Overall, the results of the current investigation show that the preheat hardening of honeybee queens during one of their immature stages is a promising method for significantly improving the productive potential of honeybee colonies. Rapid heat hardening generally refers to a rapid improvement in survival at high lethal temperatures following brief pre-exposure to a sub-lethal temperature shock [10,11]. This study found that honeybee colonies headed by heat-treated queens (pH-TQs) and non-treated queens (nH-TQs) exhibited a triple cycle of brood rearing and adult populations within a year. The three peaks of the brood cells observed in March, May, and October of each year were comparable to those of the stored pollen inside the experimental colonies. The pollen foraging tendency of honeybee colonies depends on the genotype, seasonal availability, number of larvae, and amount of stored pollen [40,41]. The activity of pollen foragers depends on the flowering time of the plant species and the outside temperature [42]. A previous study found that an ambient temperature of 20 °C stimulated the highest foraging activity, while an ambient temperature of 43 °C resulted in the lowest activity [43]. Flying outside the hive under elevated temperatures may alter foraging behavior [44]. In addition to facing heat stress during their foraging activities, bees might also suffer from problems with their thorax, which is crucial for their ability to fly. Honeybee workers show prolonged flight only with thorax temperatures between 29 and 49 °C [45,46].

### 4.2. Effect of Preheat Hardening on Brood Numbers

For honeybees to successfully grow, reproduce, and build strong colonies, they must have access to enough flowering plant resources at the right time [47]. Honeybee foragers collect nectar, pollen, and water from flowering plants [48]. Importantly, pollen grains are the sole nitrogenous source of food for brood rearing, normal development, worker longevity, and sustaining the requirements of other members of the hive [49,50]. Here, the observed decline in brood rearing for a short period in April can be explained by the reduced foraging activities of the worker bees, and these can be attributed to the local high temperatures, the seasonal east sandstorm event, the low relative humidity from the end of March to April in the Jordan Valley, and fewer places for egg laying due to the storage of honey in the brood areas inside the hives. The second reduction in brood rearing was recorded starting from June, and it was presumably due to the abundant incoming nectar and pollen deposited in the brood area after transporting the bee colonies to the upland areas of Jordan. This activity may have restricted the number of cells available to the queen for ovipositing. A reasonable gradual decrease in incoming nectar and pollen was expected during the hot, summer period from July to September, with a simultaneous rapid decrease in brood-rearing activities. The third reduction in brood rearing was observed in November with a brood stop; this was probably because of the prevailing ambient cold conditions preventing the workers from flying effectively outside the hives, as well as the reduced flowering of wild and cultivated plants. These observations are in alignment with those previously reported under semiarid conditions in the same study area [51] and in other parts of the world [52].

### 4.3. Changes Induced by Preheat Hardening on Honeybee Workers

In this study, both experimental honeybee colonies headed by the pH-TQs and nH-TQs were subjected to the same environmental conditions of the mild spring months and hot dry weather conditions prevailing during the summers in Jordan. Similarly to other colonies, the workers in both groups developed mechanisms to regulate their thoracic temperature, prevent overheating, and remain active across wide ranges of air temperatures [53]. During the summer and later months from May to the end of the year, the colonies headed by the pH-TQs built significantly more brood areas, produced more workers, and collected more pollen than the colonies headed by the nH-TQs. These differences could be due to differences in innate mechanisms, such as changes in the expression of heat shock proteins (HSPs), an increased rate of heat loss, and a decreased rate of metabolic heat synthesis to prevent thorax overheating [53,54]. Moreover, the increased productivity of the pH-TQ colonies compared to that of the nH-TQ colonies may be attributed to the rapid synthesis of heat resistance substances, such as mannitol and sorbitol, both of which have been linked to increased heat tolerance in insects [55,56,57]. Any or all of these factors could explain the improvement in the natural foraging activities of the workers in the colonies headed by the preheated queens (pH-TQs), consequently increasing the amounts of nectar and pollen collected during the hot months and until the end of the year. This increased activity of pollen collection probably led to the increase in brood rearing and, subsequently, the increase in the worker population.

Previous studies have shown that rapid heat hardening in animals during the immature stages results in the production of individuals with improved body characteristics, represented by a larger body size, weight, and body organs [58,59,60]. These changes are further expressed in changes in the performance of the subsequent adult stages [61,62]. Examples of the effects of rapid heat hardening on the body morphology of insects are as follows: an increase in the body size of the ground cricket *Allonemobius socius*, the leafhopper *Scaphoideus titanus*, and the speckled wood butterfly (*Parage aegeria*) [62,63]; an increase in the body weight of *Harmonia axyridis* [64]; and an increase in the wing size of *Drosophila mercatorum* and the hoverfly *Eristalis arbustorum* [65,66]. The changes in the body morphology of worker honeybees due to heat hardening are currently under investigation by the authors.

The increase in the body size of insects may contribute to better heat tolerance and therefore increased productivity. In ants, an increase in the body size of workers can significantly improve their heat tolerance [67,68,69]. In bees, larger workers can reach flowering resources more quickly and over a greater distance than their smaller counterparts, thereby resulting in a better foraging scope and a higher pollen load [70,71]. Foraging for long distances with a better adaptability to hot weather conditions can explain the currently observed improved pollen collection of the pH-MQs. Furthermore, such an increased food supply inside the hive can accelerate the queens’ egg-laying activity, thereby resulting in a larger worker population. Moreover, in accordance with metabolic scaling theory [72], the energy efficiency of insect flight increases with body mass [73,74,75], with larger insects exhibiting different flight kinematics expressed in different wingbeat frequencies [74,75]. Another important consideration here is the capacity of individuals to store food. Across insects, the absolute energy demand for flight is generally greater in larger-bodied individuals, which can be compensated for by greater baseline endogenous energy stores. This results in an increased flight endurance capacity with an increasing insect body size [76].

Resistance to extremely high ambient temperatures is achieved by a combination of qualities strongly affected by a prior-experienced environment [77]. Preheat hardening results in the enhanced expression of several heat shock proteins (HSPs) [56,78,79]. The expression of HSPs was found to be significantly associated with improved heat resistance [80,81] and might help prolong life [82], hence rendering the bees more tolerant to heat changes and better adapted to increased ambient temperatures. Additionally, preheat hardening was previously linked to the induction of immune gene expression [83], which might enhance the insects’ responses to parasitic pathogens and stresses or unfavorable conditions [84]. Such an increase in the tolerance of bees might further lead to better flight foraging frequencies outside the hive. Additionally, previous studies showed that preheat hardening induces the expression of oxidative stress genes that diminish the adverse effects of free radicals, such as reactive oxygen species (ROS) [85,86]. The cellular scavenging of ROS was previously linked to a reduction in the oxidative stress imposed on honeybees, which was further linked to an increase in the lifespan of both honeybee workers and queens [87,88].

## 5. Conclusions

The current results show that exposing mother queens to non-lethal heat shock has positive effects on their reproduction and further improves the resilience of their worker daughters to environmental stressors. The increased ability of honeybee workers to collect nectar and pollen during the hot summer months indicates that they have a higher tolerance to ambient heat and allows them to overcome food shortages, which may compensate for the ongoing decline in bee populations locally and globally. In the context of beekeeping, we recommend that beekeepers expose honeybee queens to preheat hardening in their immature stages as a method to improve their subsequent daughter workers. This could be an effective way to maintain beehives and improve the heat tolerance of treated bees. Future studies examining the impact of preheat hardening on honeybee immunity and other factors involved in improving honeybee tolerance and adaptation to different stress conditions are recommended.

## Data Availability

The original data presented in the study are openly available in https://drive.google.com/drive/folders/1o62SHEW9xL5OBC6ZxBEIT4BEi5fjOMkh?usp=sharing, (accessed on 22 May 2025).

## Supplementary Materials

The following supporting information can be downloaded at: https://www.mdpi.com/article/10.3390/insects16080858/s1, Table S1: Repeated Measures ANOVA Results for Hive Parameters (2021–2022). TM: thermally manipulated.

## Abbreviation

The following abbreviations are used in this manuscript:

pH-TQ	Pre-heat-treated queens
nH-TQ	non-heat-treated queens
RH	Relative humidity
