# Supplementary material for: Assessing the Productivity of Colonies Headed by Preheated Honeybee Queens
Source: Insects. 2025 Aug 18;16(8):858. doi: 10.3390/insects16080858 (PMC12386842; doi:10.3390/insects16080858)
Supplement: Supplementary file 1 [file insects-16-00858-s001.zip › insects-3738455-supplementary.pdf]

Supplementary Table S1: Repeated Measures ANOVA Results for Hive Parameters (2021–2022). TM: thermally manipulated.

Parameter Name	Year	Effect Type	Degrees of Freedom	Mean Square Error (MSE)	F Statistic	Generalized Eta-Squared	p-value
Number of brood cells	2,021	TM	1, 22	18,035,224.12	147.10	0.697	<.001
Number of brood cells	2,021	Month	11, 242	3,118,770.95	496.77	0.937	<.001
Number of brood cells	2,021	TM:Month	11, 242	3,118,770.95	16.19	0.325	<.001
Number of brood cells	2,022	TM	1, 22	21,751,635.10	177.87	0.780	<.001
Number of brood cells	2,022	Month	11, 242	2,537,220.50	538.05	0.932	<.001
Number of brood cells	2,022	TM:Month	11, 242	2,537,220.50	31.36	0.445	<.001
Monthly changes in worker numbers	2,021	TM	1, 22	35,612,635.73	47.68	0.525	<.001
Monthly changes in worker numbers	2,021	Month	11, 242	3,111,974.58	423.27	0.904	<.001
Monthly changes in worker numbers	2,021	TM:Month	11, 242	3,111,974.58	13.65	0.233	<.001
Monthly changes in worker numbers	2,022	TM	1, 22	21,137,709.72	128.53	0.687	<.001
Monthly changes in worker numbers	2,022	Month	11, 242	3,202,149.39	277.26	0.887	<.001
Monthly changes in worker numbers	2,022	TM:Month	11, 242	3,202,149.39	16.02	0.313	<.001
Drone brood cells	2,021	TM	1, 22	107,880.71	102.13	0.484	<.001
Drone brood cells	2,021	Month	11, 242	38,668.68	409.82	0.937	<.001
Drone brood cells	2,021	TM:Month	11, 242	38,668.68	42.50	0.606	<.001
Drone brood cells	2,022	TM	1, 22	102,888.22	53.90	0.297	<.001
Drone brood cells	2,022	Month	11, 242	44,977.08	214.30	0.890	<.001
Drone brood cells	2,022	TM:Month	11, 242	44,977.08	15.35	0.366	<.001
Number SWARM QUEEN CELLS	2,021	TM	1, 22	83.99	139.81	0.468	<.001
Number SWARM QUEEN CELLS	2,021	Month	11, 242	47.61	433.00	0.944	<.001
Number SWARM QUEEN CELLS	2,021	TM:Month	11, 242	47.61	57.02	0.691	<.001
Number SWARM QUEEN CELLS	2,022	TM	1, 22	138.76	89.19	0.452	<.001
Number SWARM QUEEN CELLS	2,022	Month	11, 242	49.27	372.88	0.931	<.001
Number SWARM QUEEN CELLS	2,022	TM:Month	11, 242	49.27	61.60	0.690	<.001

Parameter Name	Year	Effect Type	Degrees of Freedom	Mean Square Error (MSE)	F Statistic	Generalized Eta-Squared	p-value
Number POLLEN STORED CELLS	2,021	TM	1, 22	11,042,005.15	64.93	0.665	<.001
Number POLLEN STORED CELLS	2,021	Month	11, 242	487,803.42	687.60	0.911	<.001
Number POLLEN STORED CELLS	2,021	TM:Month	11, 242	487,803.42	27.65	0.291	<.001
Number POLLEN STORED CELLS	2,022	TM	1, 22	16,026,170.10	76.74	0.626	<.001
Number POLLEN STORED CELLS	2,022	Month	11, 242	1,578,168.36	276.38	0.867	<.001
Number POLLEN STORED CELLS	2,022	TM:Month	11, 242	1,578,168.36	16.30	0.278	<.001
Flight time in hours	2,021	TM	1, 22	185.36	23.96	0.470	<.001
Flight time in hours	2,021	Time	11, 242	3.81	597.37	0.834	<.001
Flight time in hours	2,021	TM:Time	11, 242	3.81	35.92	0.232	<.001
Flight time in hours	2,022	TM	1, 22	137.02	20.25	0.427	<.001
Flight time in hours	2,022	Time	11, 242	2.95	848.37	0.881	<.001
Flight time in hours	2,022	TM:Time	11, 242	2.95	34.04	0.229	<.001
Monthly pollen foragers	2,021	TM	1, 22	404.41	31.88	0.428	<.001
Monthly pollen foragers	2,021	Month	11, 242	34.49	253.04	0.848	<.001
Monthly pollen foragers	2,021	TM:Month	11, 242	34.49	13.22	0.225	<.001
Monthly pollen foragers	2,022	TM	1, 22	676.75	27.69	0.444	<.001
Monthly pollen foragers	2,022	Month	11, 242	35.30	231.02	0.793	<.001
Monthly pollen foragers	2,022	TM:Month	11, 242	35.30	16.39	0.214	<.001
Pollen load weight	2,021	TM	1, 22	2,105.40	48.05	0.596	<.001
Pollen load weight	2,021	Month	11, 242	92.39	343.85	0.836	<.001
Pollen load weight	2,021	TM:Month	11, 242	92.39	34.00	0.335	<.001
Pollen load weight	2,022	TM	1, 22	2,015.64	33.88	0.544	<.001
Pollen load weight	2,022	Month	11, 242	53.74	480.19	0.832	<.001
Pollen load weight	2,022	TM:Month	11, 242	53.74	15.45	0.137	<.001
